# 11010 The diagnostic accuracy of procalcitonin for urinary tract infection in hospitalized older adults

**DOI:** 10.1017/cts.2021.477

**Published:** 2021-03-30

**Authors:** Justin Choi, Kerry Meltzer, Anna Cornelius-Schecter, Assem Jabri, Matthew Simon, Matthew McCarthy, Lars Westblade, Saurabh Mehta, Zhen Zhao, Marshall Glesby

**Affiliations:** 1 Weill Cornell Medicine; 2 Cornell University

## Abstract

ABSTRACT IMPACT: This work seeks to improve the diagnostic accuracy of urinary tract infection among hospitalized older adults and mitigate antibiotic overuse in this population. OBJECTIVES/GOALS: Primary objective: To determine the diagnostic accuracy of serum procalcitonin (PCT) for the diagnosis of symptomatic urinary tract infection (UTI) in hospitalized older adults. Secondary objectives: (1) To develop a predictive model for the diagnosis of UTI; (2) To determine the ability of PCT in discriminating between lower and upper UTI. METHODS/STUDY POPULATION: We performed a prospective observational cohort study of 228 participants from a single institution. The study population included older adults (age 65 or older) who were hospitalized on the general medicine wards with a possible or suspected urinary tract infection (UTI). Upon obtaining informed consent, serum procalcitonin (PCT) was processed on remnant blood samples collected from the emergency department. We performed additional data collection through the electronic health record to obtain demographic information, clinical characteristics, and other laboratory and imaging results. Clinicians were surveyed for the diagnosis of UTI and charts were adjudicated by independent reviews of the medical record by infectious diseases experts to determine the primary endpoint of symptomatic UTI. RESULTS/ANTICIPATED RESULTS: We anticipate that serum procalcitonin predicts the presence of symptomatic urinary tract infection (UTI) by demonstrating an area under the receiver operating characteristic curve of at least 0.85. A predictive model developed in our cohort for the diagnosis of symptomatic UTI will be improved by the addition of serum PCT to the prediction model. Finally, we anticipate the serum PCT will accurately discriminate between upper and lower UTI. DISCUSSION/SIGNIFICANCE OF FINDINGS: Diagnosis of symptomatic UTI in hospitalized older adults is challenging and may lead to overuse of antibiotics and the development of antibiotic resistance in this vulnerable patient population. Serum procalcitonin offers a novel diagnostic strategy in the diagnosis of symptomatic UTI to enable more appropriate antibiotic therapy.

